# Morphometric and molecular insights into *Bactrocera dorsalis* (Hendel, 1912) (Diptera: Tephritidae) infestation on *Ziziphus mauritiana* Lamk. (Indian Jujube)

**DOI:** 10.3389/finsc.2026.1716183

**Published:** 2026-03-17

**Authors:** Kavin Palanivelu, Usharani Balakrishnan, Kamala Jayanthi Pagadala Damodharam, Suresh Krishnasamy, Sandeep Singh, Arul Dhayalan

**Affiliations:** 1 Department of Agricultural Entomology, Agricultural College and Research Institute, Tamil Nadu Agricultural University (TNAU), Madurai, Tamil Nadu, India; 2 Indian Council of Agricultural Research (ICAR) - Krishi Vigyan Kendra, TNAU, Aruppukottai, Tamil Nadu, India; 3 Indian Council of Agricultural Research (ICAR) - National Professor, Division of Crop Protection, Indian Institute of Horticultural Research, Bengaluru, India; 4 Indian Council of Agricultural Research (ICAR) - Krishi Vigyan Kendra, Madurai, Tamil Nadu, India; 5 Indian Council of Agricultural Research (ICAR) - All India Coordinated Research Project (AICRP) on Fruits, Department of Fruit Science, Punjab Agricultural University, Ludhiana, Punjab, India; 6 Division of Crop Protection, Indian Institute of Horticultural Research, Bengaluru, India

**Keywords:** *Bactrocera dorsalis*, ber, molecular characterization, morphometrics, phylogeny tree, Principal Component Analysis (PCA)

## Abstract

*Bactrocera dorsalis* (Diptera: Tephritidae), is an oriental fruit fly, commonly infesting fruit crops, especially Indian jujube (*Ziziphus mauritiana*) and other fruit crops in India. This study combined morphometric and molecular approaches to evaluate population variability. Eggs were creamy white, elliptical, and measured length and width of 1.30 and 0.23 mm. Mature maggots attained 7.87 and 1.94 mm, while pupae averaged 4.91 and 1.90 mm. Adults exhibited clear sexual dimorphism, with females larger (body length 6.87 mm; wing expanse 12.48 mm) than males (body length 5.74 mm; wing expanse 10.48 mm). Principal component analysis indicated that male traits such as body length and maggot size loaded strongly on the first component, while female wing and thoracic traits contributed predominantly to the second component, cumulatively explaining over 100% of the variation. Molecular characterization using COI gene sequences revealed phylogenetic clustering patterns that were consistent with the morphometric differentiation observed among populations and indicated close phylogenetic proximity of Indian populations to *B. invadens* and *B. kandiensis*. The integration of morphometric and molecular datasets thus provides a reliable framework for distinguishing populations of *B. dorsalis*, which is essential for accurate diagnostics, monitoring, and region-specific management strategies.

## 1 Introduction

Tephritid fruit flies belong to the largest group of insects in Diptera, Tephritidae, and are widely distributed worldwide, with more than 500 genera and 5,000 species (1). Among them, harmful species are mainly distributed in six genera: *Anastrepha*, *Bactrocera*, *Ceratitis*, *Zeugodacus*, and *Rhagoletis* (2). These flies are notorious, causing immense economic losses due to their ovipositional infestation in many types of fruits and vegetables (3). Moreover, these flies are highly valued quarantine or invasive agricultural pests internationally and domestically, which have rapid characteristics of spread, invasiveness, and destructiveness (4). In India, nearly three decades of information have targeted large and dangerous pest groups, such as the genera *Bactrocera*, *Carpomya*, and *Zeugodacus*. Especially, *Bactrocera dorsalis* (Hendel), *B. correcta* (Bezzi), *B. tau* (Walker), *B. zonata* (Saunders), *Carpomya vesuviana* (Costa), and *Zeugodacus cucurbitae* (Coquillett). The oriental fruit fly, *B. dorsalis* (Hendel) (Diptera; Tephritidae), is a serious pest of fruit crops in Southeast Asia, where it is endemic (5). Outbreaks of this pest are responsible for tens of millions of dollars in crop losses each year and if left undetected or unmanaged, field loss can be as high as 100% (6). *B. dorsalis* causes in mangoes that are cultivated in African regions, causes 77% damages in commercial farms of Mozambique, with financial losses at approximately $3,400 per ha (7). Ber cultivation is under threat from fruit flies, which cause yield losses of up to 80% in severe conditions (8), and they infest different species of ber in India.

In India, *B. dorsalis* is known to infest more than 60 species of fruits and vegetables in 30 families in tropical and subtropical regions of the world, and Infestations of *B. dorsalis* have been documented in more than 400 plant taxa (9). In India, *B. dorsalis* causes losses of up to 31% in mangoes (10). This includes papaya, avocado, banana, guava, mango, cherry, jujube, citrus, and chilli. Methyl eugenol (ME) is a well-known attractant for monitoring and mass trapping of *B. dorsalis* and *B. correcta* populations (11). In the case of *B. dorsalis* and other Tephritid fruit flies, detection of early stage infestation can be difficult and time-consuming, given the small size of eggs and young larvae concealed within plant tissue (12). As one of the preferred hosts for *B. dorsalis*, Indian jujube (*Ziziphus mauritiana* Lamk.) samples were collected from various regions of India. This jujube is majorly cultivated in Maharashtra, Madhya Pradesh, Andhra Pradesh, Gujarat, Rajasthan, Haryana, Punjab, Karnataka, and Tamil Nadu. Among this region cultivated, we have collected Punjab, Rajasthan, and Tamil Nadu these are the major cultivated area in India. However, some species belonging to the *Bactrocera* genus can fly for 50 km–100 km (13, 14).

In recent years, some fruit flies have expanded their distribution range and invaded India. Some populations of *B. dorsalis* have invaded several parts of India. However, Singh and Sharma (15) reported 37 insect pests infesting ber in Punjab. The first report on one of the fruit borers that exceeded a damage percentage of 10% was *Cadra cautella* (Walker) on ber in Punjab (16). According to Balikai (17), there are 22 insect and non–insect infestations in Karnataka State, out of more than 130 insect pest species in the state. Similarly, 23 species of pests in ber were recorded in Andhra Pradesh (18). Among this fruit fly, *C. vesuviana* (Costa), *B. dorsalis* (Hendal), *B. zonata* (Saunders), *B. correcta* (Bezzi) bark eating caterpillar, *Indarbela tetraonis* (Moore, *Indarbela quadrinotata* (Walker), ber butterfly, *Tarcus Theophrastus* (Fabricius), white grub, *Holotrichia consanguinea* (Blanch), and stone weevil, *Aubeus himalayanus* (Voss) are major pests (19–21). Singh (22), Singh et al. (15), Singh and Kaur (23), Singh (24), and Singh et al. (25, 26) reported several insect pests of jujube and their natural enemies in Punjab, India. Apart from these species, jujube (ber) fruits are also infested by fruit flies, *B. dorsalis* (Hendel) and *B. zonata* (Saunders) (5, 15, 24, 27) and Singh et al. (16) have reported a number of insect–pests of jujube including fruit flies, *B. dorsalis* and *B. zonata* and their natural enemies from Punjab, India. Ber fruit infestation sometimes occurs together with other fruit flies, such as *B. correcta*, *B. dorsalis*, and *B. zonata* (2, 28).

## 2 Materials and methods

### 2.1 Fruit fly survey and sample collection in different ber agro–ecosystem

The diversity of fruit flies in the ber ecosystem was observed at different locations in Tamil Nadu, such as orchards, the Regional Research Station, Aruppukottai block, Virudhunagar District (9°55°N and 78°09°E), Melur block, Madurai District (10°09°N and 78°37°E), Kallakadu, and Thoothukudi District (8°70°N and 77°86°E). Samples were collected once every 15 days from the orchards of Punjab Agricultural University (30°91°N and 75°85°E). We also received samples from the orchards ICAR–Central Institute for Arid Horticulture, Bikaner (Rajasthan) (28°N and 73°18°E) and samples from the agroforestry research station, Sardar Krushi Nagar through speed post.

#### 2.1.1 Collection and rearing of fruit flies

To record species diversity, infested fruits and fallen fruits beneath the trees were collected from the ber ecosystem and brought to the laboratory, where they were placed in sieved sand (5 cm layer) on rearing cages (45 cm × 45 cm × 45 cm). To maintain humidity in the rearing cages, water was sprinkled over the sand layer. Emerging adult fruit flies were collected from the cages and preserved in 70% ethanol for further study. To identify the fruit fly species, insect samples were sent to Dr. K.J. David, Senior Scientist (Agrl. Ento.) of the ICAR–National Bureau of Agricultural Insect Resources (NBAIR), Hebbal (Bengaluru). The species were also identified morphologically using a *Dacini* taxonomical key (29).

#### 2.1.2 Mass culturing of fruit flies

Fruit fly–infested ber fruits were collected from the orchards of the Regional Research Station (RRS), Aruppukottai, and placed in rearing cages (45 cm × 45 cm × 45 cm) containing a 5 cm layer of sieved sand to allow the maggots to pupate naturally. The sand was sprayed with water daily to maintain humidity and preserve fruit turgidity, and the cages were kept under controlled conditions (25 ± 2°C, 65%–75% RH, and 12L:12D photoperiod) throughout the rearing period. When the fruit flies started to emerge, spongy strips (2 cm × 6 cm) soaked with adult fruit fly diet (honey, protein powder, and water mixed in a 1:1:3 ratio) were kept on the walls of the cage with the help of small sticks inserted in soaked sponges and replenished regularly.

The sponges were refreshed daily and replaced every two days. Adults emerging from this rearing setup were used for morphological, morphometric, and molecular analyses. Field trapping using methyl eugenol and cue–lure traps was conducted specifically to collect male fruit flies, as these lures are male–specific attractants in Tephritid flies. Trap–caught males were used for species confirmation and molecular characterization, while females required for comparative morphometric analyses, including the collection of laboratory–oviposited eggs, were obtained exclusively by rearing from infested fruits.

### 2.2 Morphometric measurements

Ten fruit fly–infested samples were collected from each location of jujube (JFF) orchards from Regional Research Station, Virudhunagar (Tamil Nadu) and ber orchards at Punjab Agricultural University, Ludhiana (Punjab) and from the orchards of ICAR–Central Institute for Arid Horticulture (CIAH), Bikaner (Rajasthan). Adult fruit flies emerging from a total of 300 infested fruits were collected and enumerated, from which 10 individuals per location, representing approximately 17.8%–45.2% of the total emerged adult population, were randomly selected. Only specimens morphologically identified as *B. dorsalis* were subjected to detailed morphometric analysis to assess regional variations. Morphometric measurements of various body parts were performed under LEICA M205A, Software Leica Application Suite (LASX) version 4.12, and ZEISS Stemi 508 Axioplot (Carl Zeiss) software ZAS stereoscopic microscope fitted with a camera at the Centre for Plant Protection Studies, Tamil Nadu Agriculture University, Coimbatore, and Centre of Innovation at Agricultural College and Research Institute, Madurai. All measurements were recorded for 10 samples with an accuracy of 0.01 mm each.

### 2.3 Molecular identification

Fruit fly samples from Tamil Nadu and Punjab were subjected to molecular identification. This work was performed at the National Professor Lab at ICAR–Indian Institute of Horticultural Research, Bengaluru.

#### 2.3.1 DNA extraction

DNA was extracted using the Cetyl Trimethylammonium Bromide (CTAB) method. The samples were crushed using a pestle and mortar with sufficient liquid nitrogen. Crushed samples were transferred into 2 ml Eppendorf tubes, and 700 μl of CTAB buffer was added. The samples were incubated at 60°C for 30 min–45 min. Subsequently, 700 μl of each tube mix was carefully added and centrifuged for 10 min at 13,000 rpm. The lower detritus was removed, and the clear upper solution was transferred to a fresh 2 ml tube. A phenol:chloroform:isoamyl alcohol (25:24:1) mixture was added gradually, and the mixture was centrifuged for 10 min at 13,000 rpm. After adding an equal volume of isopropanol and sodium acetate (3M, pH 5.7) to a 1.5 ml Eppendorf tube containing the supernatant, the mixture was left overnight. The mixture was centrifuged at 13,000 rpm for 15 min, and the supernatant was discarded without disturbing the pellets. The samples were washed with ethanol (70%) and dried. The pellets were dissolved in nuclease–free water. Three microliters of RNAse were added incubated for 15 min at room temperature. Incubation was carried out at 37°C and then 55°C for 30 s and stored at −80°C (30).

#### 2.3.2 Polymerase chain reaction (PCR)

PCR was performed to amplify the sequence using the universal primer COXI–COXII to specify the region in the mitochondrial genome. The primers were prepared at a concentration of 10 pmol μl^−1^, according to the manufacturer’s instructions. The PCR reaction was carried out using a reaction mixture of 50 μl of the reaction components, 25 μl of the prepared reaction mixture (TaqDNA Polymerase Master Mix), 0.25 μl of each forward and reverse primer, 3.5 μl of water, and amplified at 48°C. The mitochondrial COI gene was amplified using the universal invertebrate primers LCO1490 and HC02198 described by (31). The forward primer LCO1490 (sequence: *5′–GGTCAACAAATCATAAAGATATTGG–3′*) had a melting temperature (Tm) of 54.80°C and produced an expected amplicon size of approximately 710 bp. The reverse primer HC02198 (sequence: *5′–TAAACTTCAGGGTGACCAAAAAATCA–3′*) had a melting temperature of 59.62°C and paired with LCO1490 to generate the standard COI barcode fragment. PCR amplification of DNA using the primers LCO1490 and HCO2198 was performed following a previously described protocol (31). The reaction began with an initial denaturation step at 95°C for 10 min, after which the samples were subjected to 35 amplification cycles. Each cycle consisted of denaturation at 94°C for 40 s, annealing at 52°C for 1 min, and extension at 72°C for 40 s. Upon completion of the cycling steps, a final extension was performed at 72°C for 40 s. The PCR products were then stored at 4°C. Amplification was performed using a thermal cycler (Techne TC–3000X ThermalCycler, UK).

#### 2.3.3 DNA sequencing

After confirming the success of DNA amplification of the targeted gene segment by electrophoresis, the samples (PCR products) were sent to a sequencing company (Eurofins Genomics India Pvt. Ltd.) to read the sequences of nitrogenous bases for each sample.

#### 2.3.4 Molecular identification of samples

Sequence processing was performed using BioEdit 7.0 (Informer Technologies, Inc.). The processed sequences were compared with the deposited sequences at the US National Center for Biotechnology Information (NCBI) using the Basic Local Alignment Search Tool (BLAST) to determine identification, which was based on the maximum score and query cover, and identify percentage provided by the research tool, highest rates of the mentioned criteria were adopted to confirm the identification to species level. After identification, the confirmed sample sequences were deposited and registered in the NCBI GenBank^®^ (https://www.ncbi.nlm.nih.gov/). Sequence homology was determined by comparing the *B. dorsalis* sequence with the NCBI GenBank database (**Table 1**).

**Table 1:** Sequence analysis results by using BLAST confirmation with US NCBI under similar accession number.

Name of species isolates	Strains	Accession number	Accession length	Query cover	E values	Percent identify	Accession length	Similarity NCBI Accession number
***Bactrocera dorsalis*** Hendal	BD1	**PQ197973** **Tamil Nadu**	660 bp	98%	0.0	98.77%	658 bp	KX259501.1 (India)
				97%	0.0	98.91%	671 bp	KX259503.1 (India)
	BD2	**PQ197976** **Punjab**	670 bp	100%	0.0	99.70%	704 bp	MN016986.1 (Tamil Nadu, India)
				100%	0.0	99.25%	696 bp	MN016995.1 (Tamil Nadu, India)
	BD	**PQ198001** **Rajasthan**	684 bp	100%	0.0	99.85%	704 bp	MN016986.1 (Tamil Nadu, India)
				100%	0.0	99.42%	696 bp	MN016995.1 (Tamil Nadu, India)

#### 2.3.5 Evolutionary relationships of taxa

The evolutionary history was inferred using the neighbor–joining method (32). The percentage of replicate trees in which the associated taxa clustered together in the bootstrap test (1,000 replicates) is shown in the branches (33). The evolutionary distances were computed using the Maximum Composite Likelihood method (34) and were in the units of the number of base substitutions per site. This analysis involved 12 nucleotide sequences. All ambiguous positions were removed from each sequence pair (pairwise deletion option). The final dataset contained 711 positions. Evolutionary analyses were conducted using MEGA11 software (35, 36).

### 2.4 Statistical analysis

All statistical analyses to determine the impact of different locations on morphometric variations and graphical representation were performed using the open–source “R” software (http://www.rstudio.com). Principal Component Analysis (PCA) for both male and female fruit flies was performed using the “Factoextra” (37) and “Factominer” (38, 39) packages. The biplot of PCA was obtained using the “ggplot2” package.

Taxonomical key characters of *Dacini* tribe

1. Face with transverse dark markings adjacent to antennal furrows, sometimes joined to form line across ventral facial margin, wing without complete costal band (Oriental)…………………………………………………………………………………………. *correcta* (Bezzi)

2. Costal band usually not expanded to form spot, abdominal tergites 3–5 always with distinct black T– shaped mark, anepisternal stripe narrow…………………………………. *dorsalis* complex

3. Wing without distinct costal band, apex of cell r4 + 5 with brown spot, thorax and abdomen pale orange………………………………………………………………………………………. *zonata* (Saunders)

**Figure 1:**
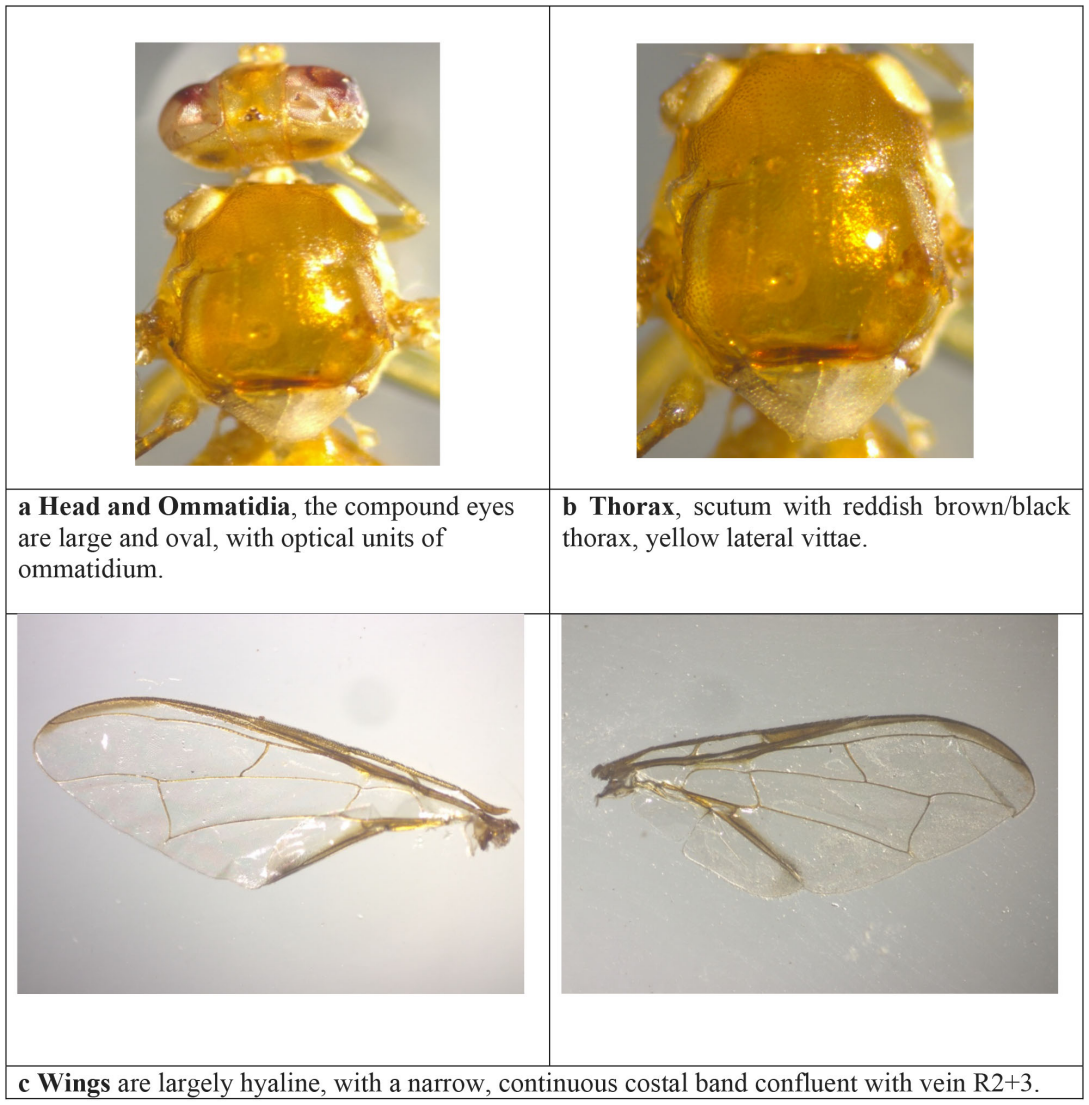
Morphological features of adult characters oriental fruit fly, *B. dorsalis* (Tephritidae, Diptera). **(a)** Head and ommatidia; the compound eyes are large and oval, with distinct optical units (ommatidia). **(b)** Thorax; the scutum is reddish brown to black, with yellow lateral vittae. **(c)** Wings largely hyaline, with a narrow, continuous costal band confluent with vein R2 + 3.

**Figure 2:**
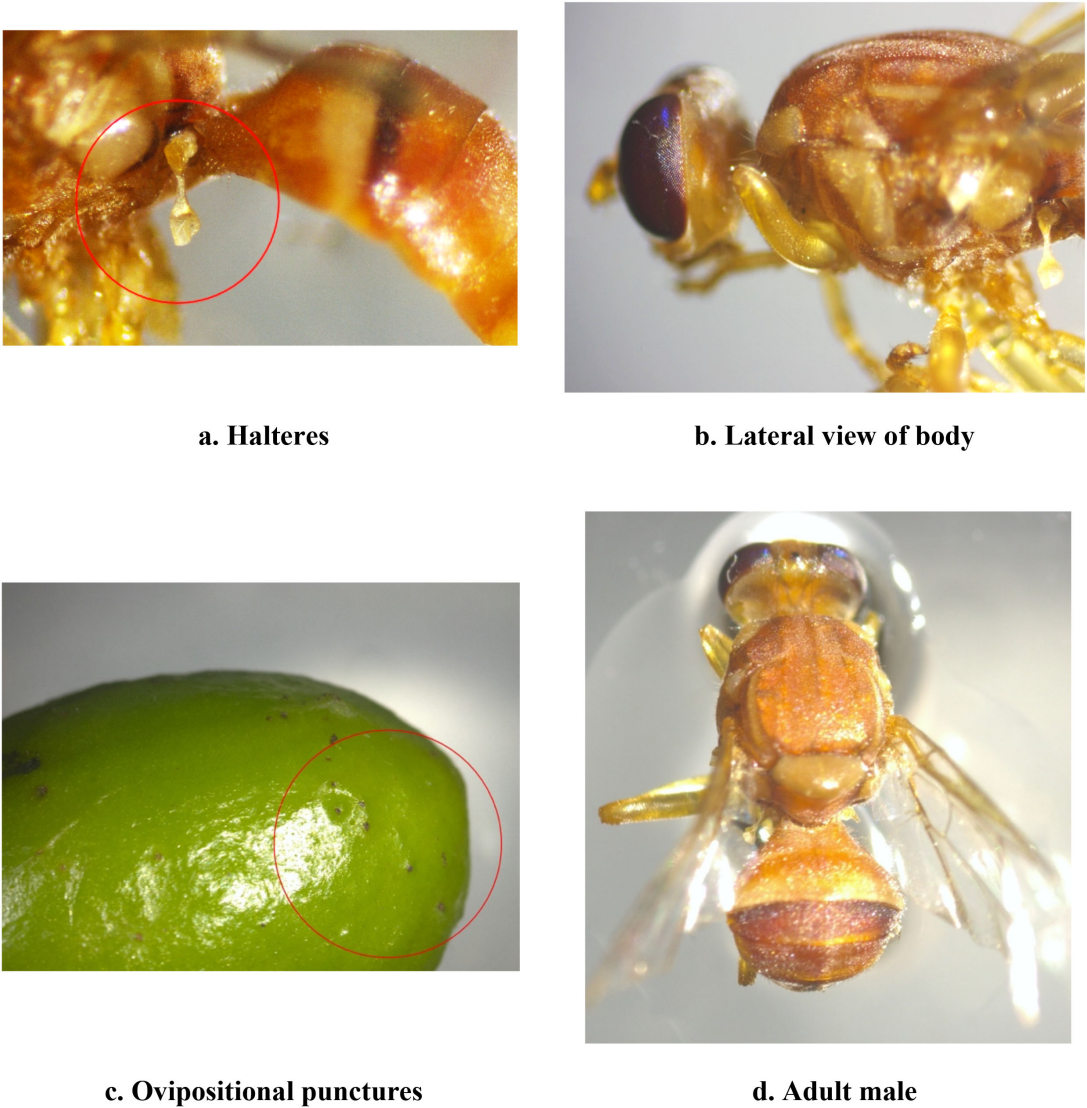
Morphological features of adult characters of guava fruit fly, *B*. *dorsalis* (Tephritidae, Diptera). **(a)** Halteres. **(b)** Lateral view of body. **(c)** Ovipositional punctures. **(d)** Adult male.

Costal band confluent with vein R2 + 3 and extending apically (at most a very slight swelling at apex of R4 + 5, pleural area ventral to postpronotal lobe pale, brown, aculeus short (1.4 mm–1.6 mm), major pest of mango, guava and many other fruits, attracted to methyl eugenol (Oriental, introduced into the Hawaiian and Mariana Islands) ……………. *dorsalis* (Hendel) (**Figures 1**, **2**).

## 3 Results

### 3.1 Morphometrics of fruit fly, *Bactrocera dorsalis* Hendel (Diptera: Tephritidae)

#### 3.1.1 Egg

The freshly laid eggs were creamy white, elliptical, and smooth, and gradually darkened as hatching approached. The anterior end of the egg bears a prominent, protruding micropylar opening, whereas the posterior end is blunt. Morphometric studies revealed that the length of eggs was 1.31 mm in Tamil Nadu, 1.28 mm in Punjab, and 1.26 mm in Rajasthan, with an overall mean of 1.30 mm. The width of eggs recorded was 0.24 mm, 0.22 mm, and 0.24 mm for the respective regions, with an overall mean of 0.23 mm (**Table 2**).

**Table 2:** Morphometrics measurements of *Bactrocera dorsalis* from different locations of India.

S. No	Characters	Tamil Nadu		Punjab		Rajasthan		Overall Mean (mm)
		Range (mm)	Mean ± Std (mm)	Range (mm)	Mean ± Std (mm)	Range (mm)	Mean ± Std (mm)	
**1**	**Egg**							
	Length	1.36–1.26	1.31 ± 0.03	1.34–1.22	1.28 ± 0.04	1.38–1.19	1.26 ± 0.06	1.30 ± 0.02
	Width	0.29–0.19	0.24 ± 0.03	0.26–0.15	0.22 ± 0.03	0.31–0.17	0.24 ± 0.06	0.23 ± 0.02
**2**	**Full grown maggots**							
	Length	7.98–7.77	7.90 ± 0.06	7.93–7.74	7.84 ± 0.06	7.98–7.78	7.87 ± 0.07	7.87 ± 0.03
	Width	2.26–1.89	2.09 ± 0.13	1.92–1.77	1.85 ± 0.05	1.97–1.79	1.88 ± 0.05	1.94 ± 0.13
**3**	**Pupa**							
	Length	5.17–4.84	4.98 ± 0.11	4.98–4.81	4.89 ± 0.06	4.96–4.79	4.87 ± 0.06	4.91 ± 0.06
	Width	2.26–1.88	2.02 ± 0.13	1.93–1.74	1.85 ± 0.05	1.88–1.76	1.82 ± 0.04	1.90 ± 0.11
**4**	**Female**							
	Length	6.96–6.77	6.87 ± 0.06	7.04–6.79	6.90 ± 0.08	6.93–6.21	6.85 ± 0.05	6.87 ± 0.03
	Wing expanse	12.59–12.39	12.48 ± 0.06	12.61–12.43	12.53 ± 0.05	12.53–12.34	12.45 ± 0.06	12.49 ± 0.04
**5**	**Male**							
	Length	5.91–5.68	5.80 ± 0.08	5.79–5.45	5.66 ± 0.11	5.88– 5.66	5.76 ± 0.07	5.74 ± 0.07
	Wing expanse	10.66–10.36	10.52 ± 0.09	10.63–10.39	10.49 ± 0.07	10.51– 10.36	10.43± 0.05	10.48 ± 0.05

*n = 10

#### 3.1.2 Maggots

The maggots were creamy white to pale, with distinct sclerites, including mandibular, suprahypostomal, and cephalopharyngeal sclerites. All larval stages were characterized by the presence of prominent mouth hooks. Upon collection, the maggots fed on fruit pulp. Morphometric observations were recorded at various developmental stages. On the fifth day after collection, the maggots appeared creamy white with a narrow anterior region and a blunt posterior region. By the eighth day, prominent sclerites were observed. The mean fully grown maggot length of RRS, APKT (Tamil Nadu), PAU (Punjab) and BKN (Rajasthan) were measured varied 7.90 mm, 7.84 mm, 7.87 mm with overall mean of 7.87 mm, respectively. The mean width of fully grown maggot length from RRS, APKT (Tamil Nadu), PAU (Punjab) and BKN (Rajasthan) were recorded that 2.09 mm, 1.85 mm, 1.88 mm and mean overall width of 1.94 mm, respectively (**Table 2**).

#### 3.1.3 Pupae

After falling on the ground, the prepupae entered the soil through wriggling movements. They pupated in the upper 4 cm layer of the soil. However, few maggots did not fall onto the ground and passed their prepupal and pupal stages in the fruits. The puparium is cylindrical, pale to dark brown, rounded at the anterior and posterior spiracles, and sometimes has distinct segmentation on the dorsal and ventral surfaces and is rounded at the posterior ends. The mean length of pupa from RRS, APKT (Tamil Nadu), PAU (Punjab) and BKN (Rajasthan) were measured varied 4.98 mm, 4.89 mm, 4.87 mm and overall mean of length 4.91 mm, respectively. The mean width of pupa from RRS, APKT (Tamil Nadu), PAU (Punjab) and BKN (Rajasthan) were recorded that 2.02 mm, 1.85 mm and 1.82 mm with overall mean width of pupa was 1.90 mm, respectively (**Table 2**).

#### 3.1.4 Adult

Adults are medium–sized with a black scutum with lateral vittae. Wings mostly hyaline, with a discontinuous costal band. Abdomen reddish–brown with a ‘T’–shaped marking. This species of *Bactrocera* is characterized by facial spots that coalesce to form a transverse line. This species is similar to *B. zonata* in having a reduced pattern on the wings and apical spots, but the costal band extends from both cells c and Sc and facial markings. Thorax, scutum predominantly black, anterior supra–alar setae present, two yellow lateral post–sutural vittae ending beyond intra–alar setae, prescutellar setae present. Scutellum completely yellow with a narrow basal black band and two apical scutellar setae (**Table 2**).

The abdomen was dark brown with a median longitudinal dark band on terga 3–5. Male with pecten on the third abdominal tergum. The body length of male and female flies from RRS, APKT (Tamil Nadu), PAU (Punjab) and BKN (Rajasthan) varied with 5.80 mm and 6.87 mm, 5.66 mm and 6.90 mm, 5.76 mm and 6.85 mm with overall mean of male and female body length measured 5.74 mm and 6.87 mm, respectively. The wings with a reduced pattern, coastal band from cell Sc to r_1_ confluent with R_2 + 3_, broken in cell r_2 + 3_, leaving an apical dark spot in cell r_2 + 3_ and r_4 + 5_ cubital streak contained with cell cup, base of cell br without my microtrichia. The morphometric wing expanse of male and female from RRS, APKT (Tamil Nadu), PAU (Punjab) and BKN (Rajasthan) were revealed with 10.52 mm and 12.48 mm, 10.49 mm and 12.53 mm, 10.43 mm and 12.45 mm, culminating in overall mean wing expanse of male and female were 10.48 mm and 12.48 mm, respectively.

#### 3.1.5 Results PCA analysis of morphometry for various stages of fruit flies

Principal component analysis (PCA) of the insect morphometric data revealed multiple components, however, the first two principal components captured nearly all the variance in the dataset. PC1 accounted for 65.681% of the variance (eigenvalue = 6.568), and PC2 accounted for 34.318% of the variance (eigenvalue = 3.431), with a combined variance of 99.999% (**Table 3**). Subsequent principal components (PC3 and beyond) contributed minimal variance (<1%) and were therefore not considered in the analysis. These results indicate that the primary variation among populations was effectively summarized by PC1 and PC2.

**Table 3:** Eigen analysis of correlation matrix in *B. dorsalis* various stages of fruit flies.

Characters	PCA1	PCA2
Eigen values	6.568	3.431
Percentage of variance	65.681	34.318
Contribution features (Variables)	Loadings	
Egg length	0.992	0.124
Egg width	0.831	−0.556
Full grown maggot length	0.998	−0.066
Full grown maggot width	0.942	0.336
Whole pupa length	0.808	0.589
Whole pupa width	0.827	0.562
Female whole body length	−0.542	0.840
Female wing expanse	−0.566	0.825
Male whole body length	0.953	−0.303
Male wing expanse	0.389	0.921

**Figure 3:**
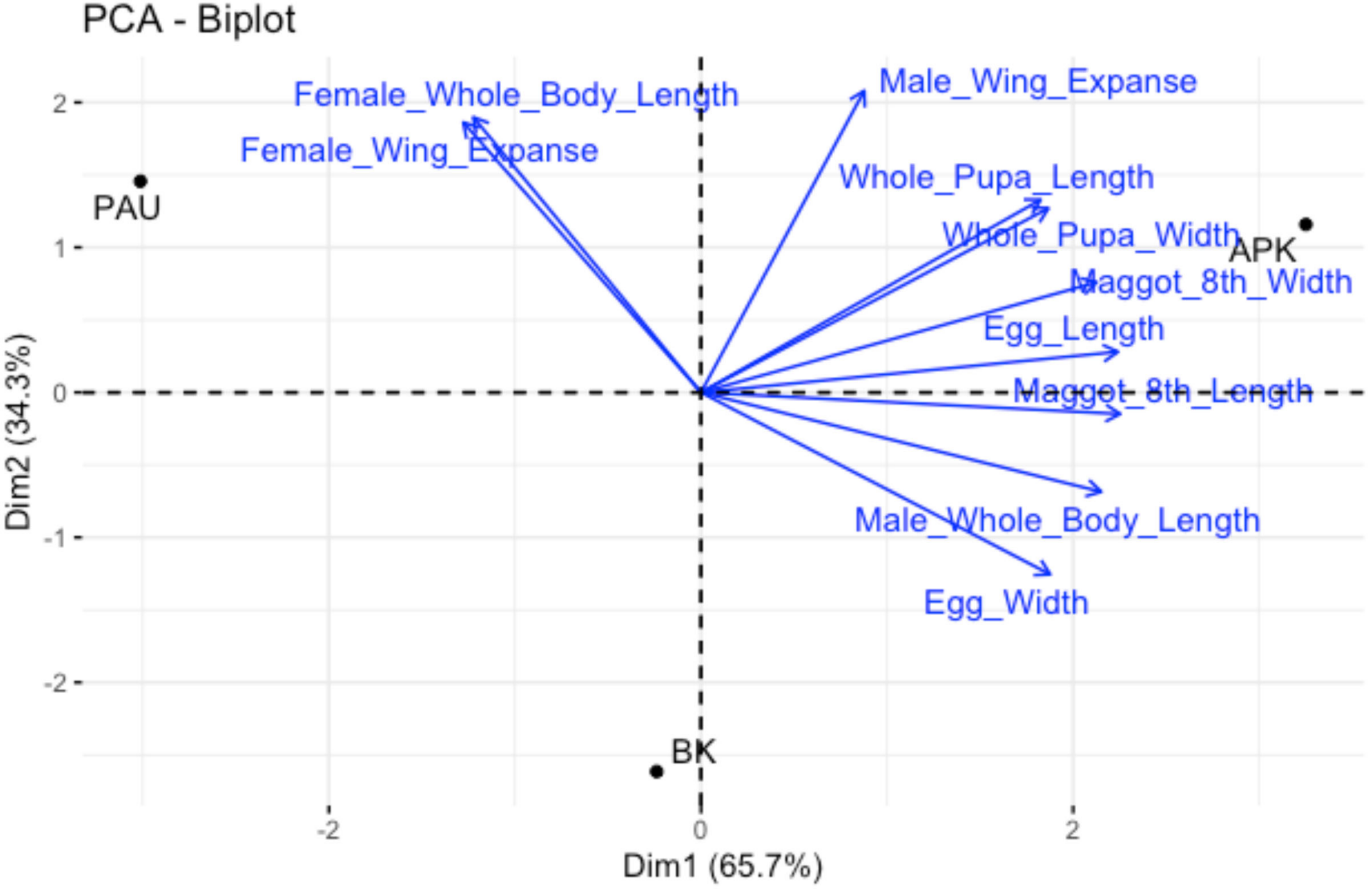
Biplot loading of the principal components showing relations among various stages of *B*. *dorsalis*.

The feature loadings on PC1 were strongly positive for most measurements, indicating a general size–related factor. Specifically, egg length (0.992), full–grown maggot length (0.998), and male whole–body length (0.953) had high, positive loadings. Egg width (0.831), full–grown maggot width (0.942), whole pupa length (0.808), and whole pupa width (0.827) also contributed positively to PC1, albeit with slightly lower values. Male wing expanse had a moderate positive loading (0.389). In PC2, the female–specific traits, including whole body length and wing expanse, displayed significant negative loadings on PC1 but strong positive loadings on PC2, at −0.542 and 0.840 for body length, and −0.566 and 0.825 for wing expanse, respectively. This suggests that female traits were differentiated along the second principal component. Conversely, male wing expanse showed the highest loading on PC2 at 0.921, indicating a distinct contribution to this component. Egg width also showed an interesting pattern, with a substantial negative loading (−0.556) on PC2, contrasting with its positive loading on PC1 (**Figure 3**).

### 3.2 Phylogenetic analysis of *B. dorsalis*

Phylogenetic analysis of *B. dorsalis* sequences, including three of my own sequences (indicated by green, red, and blue triangles), showed distinct clustering patterns with sequences from various regions, including Australia, India, Sri Lanka, Uganda, and Nigeria. The APKT (Tamil Nadu) sequence (PQ197973.1), marked with a blue triangle, clustered with *B. dorsalis* from Switzerland (DQ006864.1) and *B. invadens* from Sri Lanka (JQ627474.1), forming a well–supported clade with a bootstrap value of 43%. This indicates genetic similarity between the Aruppukottai population and the sequences from Europe and South Asia.

The sequence from BKN (Rajasathan) (PQ198001.1), marked with a red triangle, clustered with other Indian sequences, including *B. dorsalis* from PAU (Punjab) (PQ197976.1, green triangle), forming a separate subclade with a high bootstrap support of 43%. This suggests a close genetic relationship between Indian populations. Interestingly, *B. correcta* from Ludhiana (PQ197974.1) appeared as an outgroup, showing clear divergence from *B. dorsalis* sequences.

**Figure 4:**
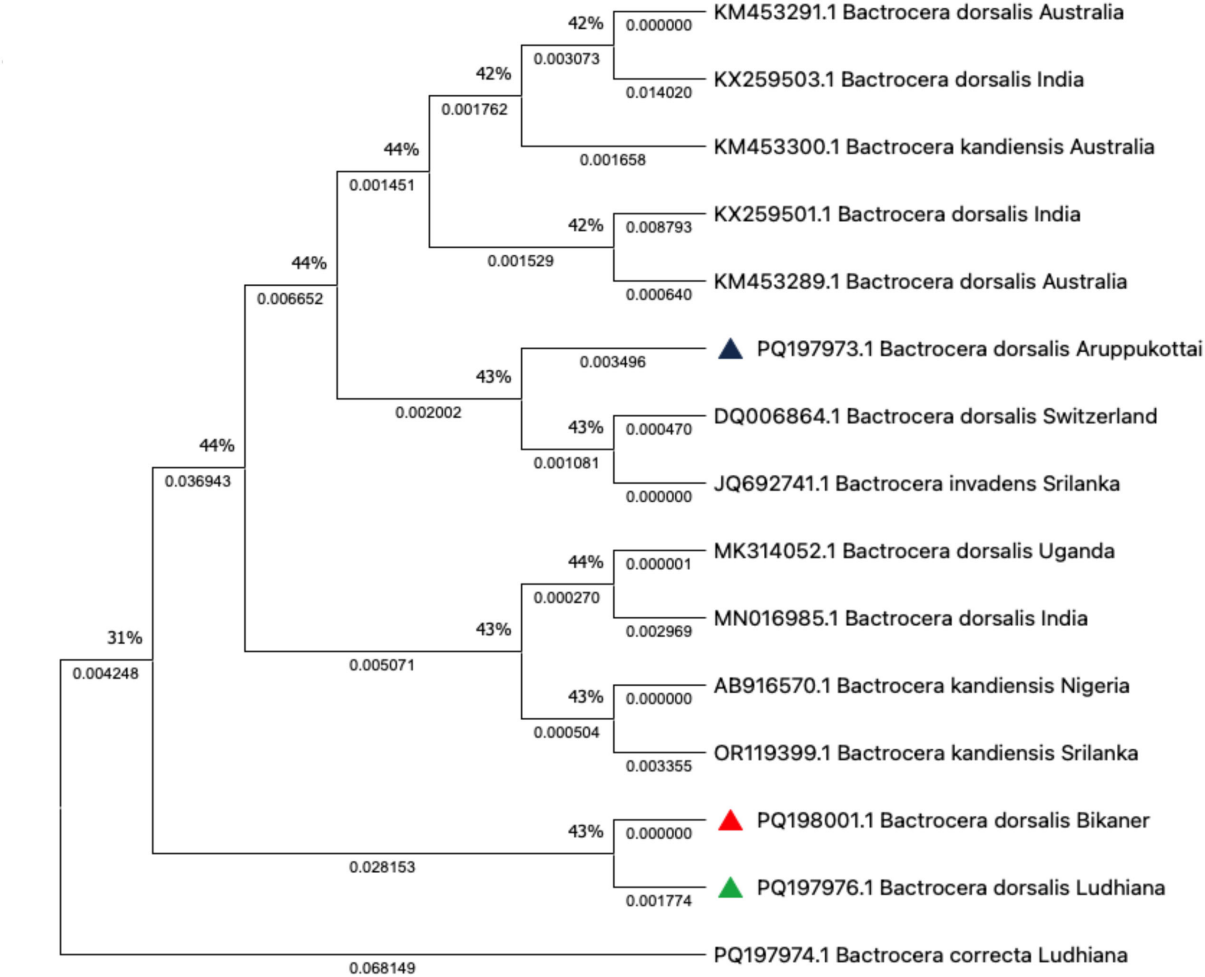
Phylogenetic tree showing its genetic relationships with other *B*. *dorsalis*.

Other clusters included *B. dorsalis* from Australia (KM453291.1, KM453298.1) and *B. kandiensis* from Australia (KM453300.1), which formed a distinct group with moderate bootstrap support. Additionally, African sequences from Uganda (MK314052.1) and India (MN016985.1) clustered with *B. kandiensis* from Sri Lanka and Nigeria (OR119939.1 and AB916570.1), suggesting genetic exchange or similarities across these regions. Overall, the tree revealed genetic relationships between populations of *B. dorsalis* and *B. kandiensis* across different geographical locations, with Indian sequences showing close clustering patterns (**Figure 4**).

## 4 Discussion

The present study provides comprehensive morphometric and molecular characterization of *B. dorsalis* populations across India, contributing to the understanding of population differentiation, phylogenetic relationships, and regional adaptation. By integrating morphometric measurements with COI gene sequencing, this study offers a robust framework for accurate species identification, which is essential for targeted monitoring and management strategies for this economically important pest.

Morphometric analysis of eggs revealed regional variation in length and width, with means ranging from 1.26 mm–1.31 mm in length and 0.22 mm–0.24 mm in width across Tamil Nadu, Punjab, and Rajasthan. These measurements are broadly consistent with earlier reports, Singh et al. (40) reported that the mean length and width of *B. dorsalis* eggs were 1.30 mm and 0.24, respectively, while Naik et al. (41) found the length and width to be 1.36 mm and 0.25 mm, respectively. The egg size of *B. dorsalis* was observed to range between 0.5 and 0.6 mm (Kalia (42), Amur et al. (43). Similarly, Leghari (44) noted that the sizes of eggs for both *B. dorsalis* and *B. zonata* varied from 0.5 mm to 0.6 mm, whereas Dale (45) observed that *B. zonata* eggs ranged from 0.75 mm to 1.01 mm and 0.16 mm to 0.25 mm, respectively.

Larval morphometrics also exhibited variation among regions. Fully grown maggots ranged from 7.84 mm–7.90 mm in length and 1.85 mm–2.09 mm in width, with means closely aligning with Kalia and Yadav (46) who reported that the mean length and width of fresh and fully grown maggots of *B. dorsalis* were 2.87 mm and 0.40 mm for fresh maggots, and 8.18 mm and 2.25 mm for fully grown maggots, respectively. In contrast, Singh et al. (40) stated that fresh maggots measured 3.80 mm in length and 0.55 mm in width, while fully grown maggots had a length and width of 8.02 mm and 1.55 mm.

Pupae similarly displayed variability in length (4.87 mm–4.98 mm) and width (1.82 mm–2.02 mm), consistent with Kalia (42), who noted the presence of a black dot on the posterior part of *B. dorsalis* pupae. Naik et al. (41) described the pupae as segmented, barrel–shaped, or cylindrical, with a color ranging from yellowish white to deep brownish yellow when newly formed. Over time, the color shifted to light brown or brownish–gray, with 11 distinct segments. The pupae measured approximately 4.08 mm in length and 1.82 mm in width. He also observed that the size of the pupae varied not only between different fruits, such as guava and mango, but also among different cultivars of mango. The present study further demonstrates that pupal morphometrics can vary not only between fruit species but also among cultivars, reinforcing the importance of population–specific baseline measurements in developmental studies. Adult morphometric traits, including body length, wing expanse, and abdominal markings, were consistent with previous observations. Kumar et al. (47) reported that the average body length of *B. correcta* was 4.71 mm for male fruit flies and 5.61 mm for females. Additionally, the average body width was 10.08 mm for males and 11.54 mm for females. Weems and Fasulo (48) noted that *B. correcta* is measured approximately 5.40 mm long. According to Manurung et al. (49), the morphometric features of *B. dorsalis* have mean body and wing lengths and wing width of 6.91 mm, 6.36 mm, and 2.43 mm, respectively. However, our PCA analysis highlighted distinct contributions of male and female traits, providing a refined framework for sex–specific identification in field populations.

Phylogenetic analysis of *Bactrocera dorsalis* revealed clustering close to the global population clade, indicating moderate genetic diversity and regional association. COI–based phylogenetic analysis provided an independent assessment of population structure and confirmed morphometric differentiation. Indian populations clustered into distinct subclades, with BKN (Rajasthan) and PAU (Punjab) sequences showing close genetic affinity, consistent with localized dispersal or ongoing gene flow. The Aruppukottai population (APKT, Tamil Nadu) clustered with European and Sri Lankan sequences, reflecting haplotype sharing that was likely driven by the anthropogenic movement of infested commodities. This observation aligns with earlier reports that *B. invadens* is conspecific with *B. dorsalis* based on mitochondrial and nuclear DNA analyses (50, 51). The modest bootstrap support (43%) observed in this clade reflects the inherent limitations of COI markers within the *B. dorsalis* complex, which often exhibits mitochondrial introgression and incomplete lineage sorting (52, 53). Such genetic overlaps are consistent with the wide dispersal and anthropogenic movement of *B. dorsalis* populations across continents, facilitated by the global fruit trade and transport of infested commodities. Therefore, the observed phylogenetic relationship is best interpreted as evidence of shared haplotypes within the *B. dorsalis* complex rather than a direct lineage link between India and Europe.

Within India, BKN (Rajasthan) (PQ198001.1) and PAU (Punjab) (PQ197976.1) sequences were clustered into a distinct subclade, substantiating the close genetic affinity among northwestern Indian populations, which might have resulted from localized dispersal or continuous gene flow within the region. The clear separation of *B. correcta* as an outgroup supports the taxonomic resolution of the COI marker for distinguishing sympatric species. The association of *B. dorsalis* and *B. kandiensis* sequences from Africa, Sri Lanka, and Australia further reflects the complex genetic plasticity, likely shaped by past introgression or limited COI resolution (50). Such inter–regional clustering patterns emphasize the need for a multilocus or integrative taxonomic approach combining mitochondrial and nuclear markers to validate species identity and trace invasion pathways (53). Expanding molecular surveillance with additional markers, such as ITS1, EF–1α, nuclear genes, and microsatellite loci, could provide greater insight into the phylogeographic structure and evolutionary dynamics of *B. dorsalis* populations across the biogeographic zones of India.

## 5 Conclusion

This study provides a comprehensive morphometric, multivariate, and molecular characterization of *B. dorsalis* populations from Tamil Nadu, Punjab, and Rajasthan. Morphometric analysis revealed clear differences across developmental stages, with females exhibiting a larger body size than males, reflecting sexual dimorphism linked to reproductive potential and dispersal ability. Principal component analysis further supported these findings, with PC1 explaining the overall size variation and PC2 capturing sex–specific differentiation, particularly in body length and wing expanse. Molecular phylogenetic analysis has highlighted considerable genetic diversity, with Indian populations clustering closely but showing affinities with Sri Lankan and European lineages, suggesting possible gene flow or shared ancestry. *B. correcta* served as a distinct outgroup, confirming species–level divergence. The integration of morphometric and molecular approaches underscores the complexity of population structuring in *B. dorsalis*, which has significant implications for its monitoring and management. Future research should expand to whole–genome sequencing and ecological niche modeling to better predict population shifts under climate change. Such multidisciplinary insights will be critical for developing region–specific, sustainable pest management strategies against this highly invasive fruit fly.

## Data Availability

The datasets presented in this study can be found in online repositories. The names of the repositories and accession numbers can be found in the article. All datasets are also publicly available in Figshare: 10.6084/m9.figshare.31402965.
