# Correction: Morphometric and molecular insights into *Bactrocera dorsalis* (Hendel, 1912) (Diptera: Tephritidae) infestation on *Ziziphus mauritiana* Lamk. (Indian Jujube)

**DOI:** 10.3389/finsc.2026.1845367

**Published:** 2026-06-10

**Authors:** Kavin Palanivelu, Usharani Balakrishnan, Kamala Jayanthi Pagadala Damodharam, Suresh Krishnasamy, Sandeep Singh, Arul Dhayalan

**Affiliations:** 1 Department of Agricultural Entomology, Agricultural College and Research Institute, Tamil Nadu Agricultural University (TNAU), Madurai, Tamil Nadu, India; 2 Indian Council of Agricultural Research (ICAR) - Krishi Vigyan Kendra, TNAU, Aruppukottai, Tamil Nadu, India; 3 Indian Council of Agricultural Research (ICAR) - National Professor, Division of Crop Protection, Indian Institute of Horticultural Research, Bengaluru, India; 4 Indian Council of Agricultural Research (ICAR) - Krishi Vigyan Kendra, Madurai, Tamil Nadu, India; 5 Indian Council of Agricultural Research (ICAR) - All India Coordinated Research Project (AICRP) on Fruits, Department of Fruit Science, Punjab Agricultural University, Ludhiana, Punjab, India; 6 Division of Crop Protection, Indian Institute of Horticultural Research, Bengaluru, India

**Keywords:** *Bactrocera dorsalis*, ber, molecular characterization, morphometrics, phylogeny tree, Principal Component Analysis (PCA)

There was a mistake in **Figure 1c** as published. The figure and its legend were incorrect. The original figure legend was:

**Figure 1:**
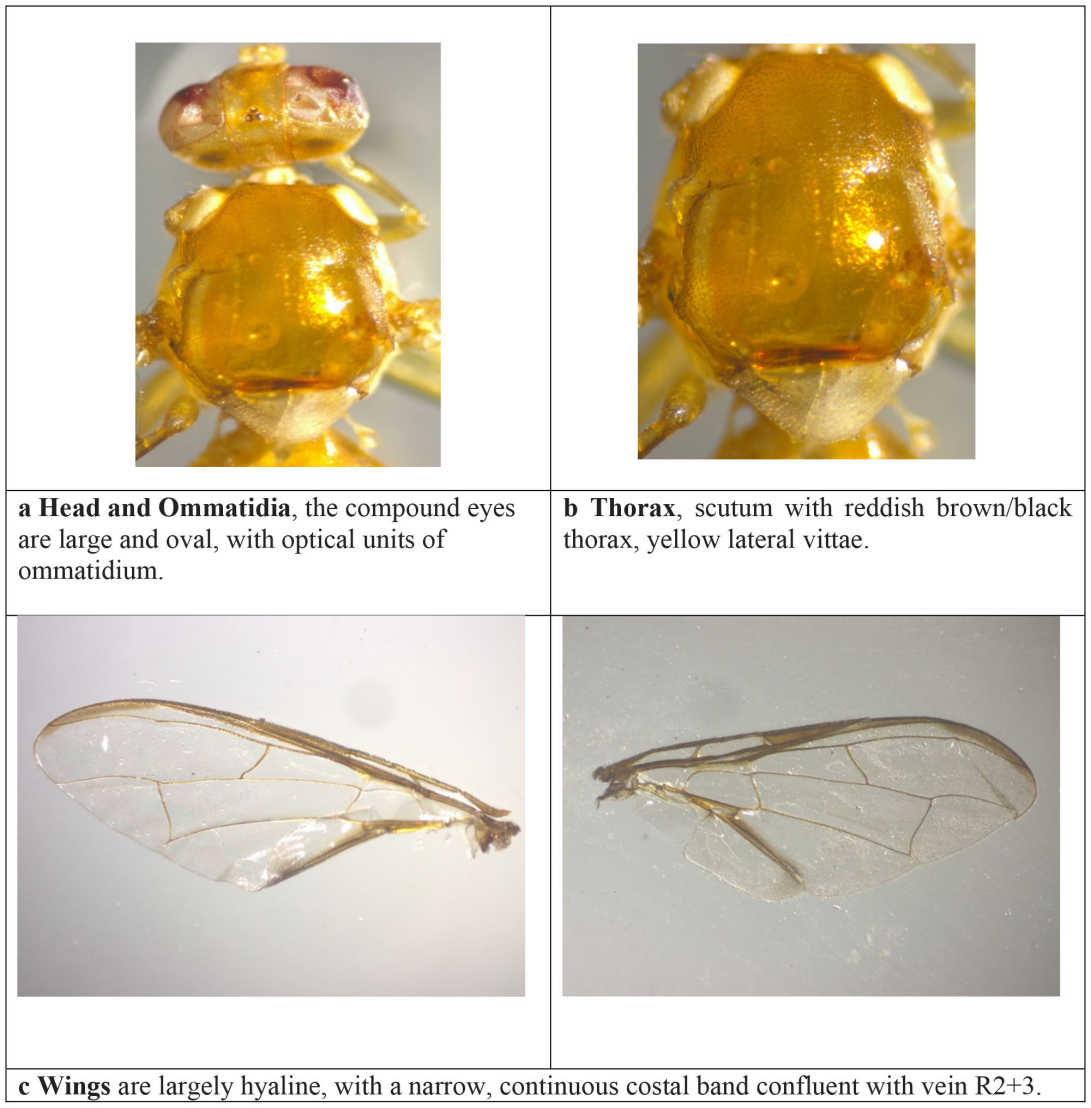
Morphological features of adult characters oriental fruit fly, B. dorsalis (Tephritidae, Diptera). **(a)** Head and ommatidia; the compound eyes are large and oval, with distinct optical units (ommatidia). **(b)** Thorax; the scutum is reddish brown to black, with yellow lateral vittae. **(c)** Wings largely hyaline, with a narrow, continuous costal band confluent with vein R2 + 3.

“Figure 1 Morphological features of adult characters oriental fruit fly, *B*. *dorsalis* (Tephritidae, Diptera). **(a)** Head and Ommatidia, the compound eyes are large and oval of optical units of ommatidium. **(b)** Thorax with anepisternum and mediotergite (below the scutellum) yellow with black spots are present on the margins of the thorax. **(c)** Wings with four yellow parallel crossband, one band occurs in region of sub basal, second one is on discal region, third origins on subapical and third and fourth bands extend toward apical margins.”

The corrected **Figure 1** and its caption appear below.

This correction does not change the scientific conclusions of the article in any way.

The original version of this article has been updated.

